# The hidden influence: Medical students’ knowledge and attitude of conflict of interest–A cross-sectional study in Jeddah, Saudi Arabia

**DOI:** 10.1371/journal.pone.0328884

**Published:** 2025-08-01

**Authors:** Ramy Samargandi, Rawad H. Alsayed, Mazen A. Alqarni, Sohail S. Alghamdi, Adel Alzahrani, Anas Almutairi, Safinaz M. Alshiakh, Abdullah S. Algarni

**Affiliations:** 1 Department of Surgery, College of Medicine, University of Jeddah, Jeddah, Saudi Arabia; 2 College of Medicine, University of Jeddah, Jeddah, Saudi Arabia; 3 Department of Emergency Medicine, Faculty of Medicine, King Abdulaziz University, Jeddah, Saudi Arabia; 4 Department of Medicine, College of Medicine, University of Jeddah, Jeddah, Saudi Arabia; Shahid Beheshti University of Medical Sciences, IRAN, ISLAMIC REPUBLIC OF

## Abstract

**Introduction:**

Conflicts of interest (COI) pose ethical challenges in medical education and clinical practice, potentially influencing decision-making. While COI policies and education vary globally, inconsistent training leaves medical students vulnerable to industry influence. In Saudi Arabia, COI education remains underexplored. This study assesses medical students’ knowledge, attitudes, and exposure to COI, aiming to identify gaps and inform educational improvements.

**Methodology:**

A cross-sectional study was conducted among 392 medical students from multiple universities in Jeddah, Saudi Arabia. A validated questionnaire was administered to assess COI knowledge, attitudes, and exposure to industry interactions. Data were analyzed using IBM SPSS 29.0, with statistical significance set at p < 0.05.

**Results:**

A total of 392 participants were included. Overall, 71.4% of students were able to define COI, while 28.6% lacked awareness. Clinical students had significantly higher knowledge scores than preclinical students (p = 0.001). Grade Point Average (GPA) was significantly associated with attitudes toward COI, with students who had excellent GPAs scoring the highest (p < 0.001). No significant differences were observed in knowledge or attitude scores based on gender, research experience, and university affiliation. Over half of the students (52.6%) felt inadequately educated about COI, and 48.5% had never attended a COI lecture. Clinical students (65.2%) reported more interactions with pharmaceutical representatives than preclinical students (34.8%).

**Conclusion:**

While medical students generally recognize COI, gaps in knowledge and formal education persist. Clinical exposure appears to enhance knowledge, yet inconsistent education leaves students vulnerable to industry influence. Strengthening COI education within medical curricula is essential to promote ethical decision-making and uphold professional integrity.

## Introduction

Conflicts of interest (COI) are described as a collection of circumstances that creates a risk of professional judgement about a main interest (e.g., patient care) being excessively impacted by a secondary interest (e.g., financial benefit) [1]. Medical students can be seen as potential trophy targets for marketing by pharmaceutical companies, as they are often offered gifts or free courses to influence their future prescribing habits. During the clinical years, students interact with pharmaceutical companies, putting them at risk of COI without adequate education on the subject. This lack of awareness makes them more susceptible to marketing strategies. Many studies highlight the importance of teaching COI early on to ensure that medical students can recognize and manage these conflicts effectively [2].

In 2016, 80% of interns in Japan had attended sponsored seminars, and over 98% received gifts from pharmaceutical companies, such as notepads or dinners. When they first started their training, interns had far more interactions with pharmaceutical companies than students did [3].

In 2007, the American Medical Student Association (AMSA) began tracking medical schools’ COI policies, introducing a detailed scorecard in 2008. Initially, most schools scored poorly, but the percentage earning A grades rose from 4.7% in 2008 to 25.9% by 2013, marking a significant policy shift in less than a decade [4]. This led to similar efforts in Australia [5], Canada [6], and France [7]. In Australia, a study revealed that 7 out of 20 medical schools had a COI policy, while in Canada, 16 out of 17 medical schools had such policies. On the other hand, the situation in France was quite different, with only 2 out of 37 faculties had COI policies [7], not far from France, 40% of faculties in Belgium have COI policies; however, these policies are weak, vague, and more like recommendations than strict rules [8]. In Germany, 90% of students say their curriculum doesn’t cover interactions with pharmaceutical companies. Over half (65%) feel unprepared for these interactions, and 60% want additional training on the topic [9].

The Ministry of Health in Saudi Arabia has actively pursued COI policies to ensure transparency and integrity within the healthcare system. By the end of 2018, the Ministry implemented a comprehensive COI disclosure policy in accordance with the Council of Ministers’ decision, as outlined in the Code of Conduct and Public Service Ethics, particularly in Chapter Five, “Conflict of Interest and Anti-Corruption” [10]. Despite these clear policies and efforts at the national level, the situation within medical schools remains less understood. Previous studies evaluating medical students’ knowledge regarding COI in Korea and France showed inadequate levels of knowledge and understanding of COI. Both studies demonstrated that students had not received sufficient education on this important topic [11,12]. To date, no studies have evaluated the knowledge and attitudes of medical students toward COI in Saudi Arabia. This research aims to explore the current level of understanding and attitudes toward COI among medical students and identify the factors influencing their knowledge and attitudes.

## Materials and methods

This cross-sectional study aimed to evaluate the level of knowledge and attitudes toward COI among medical students across various universities in Jeddah, Saudi Arabia. Ethical approval was granted by the Jeddah University Bioethics Committee of Scientific and Medical Research (Number: UJ-REC-273). Jeddah is a major urban city in Saudi Arabia with a population exceeding four million. It hosts several public and private medical institutions. The city’s diverse academic landscape draws students from various regions and socioeconomic backgrounds across the country. The study included medical students from all academic years, both preclinical (2nd and 3rd year) and clinical (4th, 5th, and 6th year), from universities in Jeddah who provided written informed consent. Participation involved reading an information script and agreeing by clicking a link to complete the questionnaire. Pre-medical (1st-year) students, non-medical students, individuals not affiliated with universities in Jeddah, and those who declined to participate were excluded.

Data were collected from January to March 2025 using a previously validated questionnaire that has been used in international studies [11,12], and permission was obtained from the original authors for its use [11]. The questionnaire consisted of three sections including sociodemographic information, knowledge and attitudes toward COI, and exposure to pharmaceutical industry interactions and perceived consequences of COI. Minor modifications were made to adapt the questionnaire to the cultural and contextual setting of Jeddah, while preserving its original structure to enable comparison with international studies. To establish face validity, a panel of three experts in medical education evaluated the questionnaire’s content, structure, and language to ensure that the items were relevant, comprehensive, and appropriate for the study objectives. Their feedback was incorporated into the final version. Additionally, a pilot test with 20 medical students was conducted to assess clarity and feasibility from the perspective of the target population. The complete version of the survey questionnaire used in this study is available in (S1 Text). A convenience sampling technique was employed, and data were gathered from both private and governmental medical institutions in Jeddah, Saudi Arabia. The questionnaire was administered through Google Forms (Alphabet Inc., Mountain View, CA) via social media platforms, ensuring convenient, anonymous, and voluntary participation across multiple universities. The sample size was calculated using the Raosoft® online sample calculator [13]. The calculation was based on a 95% confidence level, 5% margin of error, and 50% response rate. The estimated population size across all universities was approximately 6000 students. The minimum required sample size to ensure representativeness was determined to be 362 participants.

Statistical analysis was conducted on the dataset, encompassing both descriptive and inferential methodologies. A descriptive analysis is conducted to summarize the demographic characteristics of the participants, which include age, gender, and other features. Descriptive statistics were presented as counts, proportions (%), medians, and means with standard deviations, as appropriate. A scoring system was applied in which one point was awarded for each correct answer. The knowledge domain included 11 multiple-choice questions (Yes/No/I don’t know), and the attitude domain included 9 similarly structured items assessing ethical reasoning and attitudes. For both domains, a “Yes” response indicating a correct or ethically appropriate answer was scored as 1, while “No” and “I don’t know” were scored as 0.

To assess the sociodemographic factors that may influence participants’ knowledge and attitudes toward COI, statistical analyses were performed, including the Chi-Square test for associations between categorical variables and the Independent T-test and ANOVA for associations between continuous variables. A significance level of p < 0.05 was considered statistically significant. All statistical analyses were conducted using the Statistical Package for the Social Sciences (SPSS), version 29.

## Results

### Participant demographics

**Fig 1:**
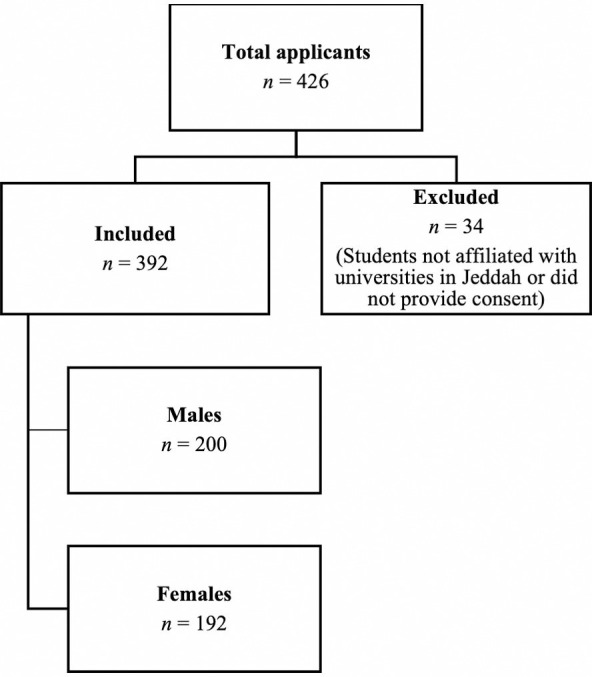
Flowchart of the participants.

A total of 426 participants initially responded to the questionnaire. Of these, 34 were excluded from the study, including 18 who refused to participate and 16 who were from outside Jeddah. Consequently, the final sample comprised 392 participants (Fig 1). The sample included 192 females (49.0%) and 200 males (51.0%). The majority were aged between 18 and 23 years, accounting for 311 students (79.3%), with a mean age of 22.1 years ± 1.9. Participants were primarily affiliated with the University of Jeddah (49.0%) and King Abdulaziz University (29.1%). In terms of education level, 284 students (72.4%) were in the clinical phase, while 108 (27.6%) were in the preclinical phase. More than half of the participants (*n* = 206, 52.6%) reported an excellent Grade Point Average (GPA), and 240 (61.2%) had no prior research experience (Table 1).

**Table 1:** Sociodemographic other parameters of all participants (*n* = 392).

		Frequency n (%)
**Gender**	Female	192 (49.0)
	Male	200 (51.0)
**Age**	18–23 Years	311 (79.3)
	24–30 Years	81 (20.7)
	Mean (Sd)	22.1 (±1.9)
	Range	18-30
**University**	University of Jeddah	192 (49.0)
	King Abdul Aziz University	114 (29.1)
	King Saud University	20 (5.1)
	Private Universities*	66 (16.8)
**Stage of Education**	Preclinical	108 (27.6)
	Clinical	284 (72.4)
**GPA**	Poor	2 (0.5)
	Average	26 (6.6)
	Good	158 (40.3)
	Excellent	206 (52.6)
**Research Publications**	No Previous Research	240 (61.2)
	1–3	123 (31.4)
	4–6	17 (4.3)
	7–10	6 (1.5)
	More than 10	6 (1.5)

*Private Universities: (Ibn-e-Sina, Batterjee, Fakeeh Universities), GPA: Grade Point Average.

### Knowledge and attitudes toward COI

The majority of participants (71%) could define COI, while 28.6% lacked awareness. Perceptions of specific COI scenarios varied, with 58% not considering minor gifts a conflict, whereas 42% did. Similarly, 53% viewed having a close relative in the pharmaceutical industry as a COI, while 47% disagreed. Nearly half of the participants considered lunch or dinner invitations a COI. Recognition of conflicts was higher for industry-sponsored clinical study participation (57%), receiving a fellowship (54%), being paid as a speaker (58%), holding stock (61%), and receiving salary or honoraria (64%). In terms of education, 53% reported inadequate COI instruction, 49% had never attended a COI lecture, and 63% had not researched COI independently. While 77% wanted teachers to disclose their COI, only 40% reported such disclosures occurring. Over half of the participants (55.4%) felt inadequately educated on COI in the medical field. Most participants agreed that medical schools should play a role in guiding student interactions with industry representatives (84%). Regarding COI education in the curriculum, 80.9% of participants supported integrating COI as a subject in medical school. Furthermore, 82.4% expressed interest in receiving more information about COI (Table 2)*.* For source of information, 78.1% of participants preferred lectures or tutorials as their primary source of COI information. Other preferred sources included information provided outside the faculty, such as conferences or meetings (41.8%), and courses integrated into Critical Appraisal of Articles (41.1%). Additionally, 29.3% of participants favored receiving written documents, while 19.9% preferred newsletters or mailing lists.

**Table 2:** Knowledge and attitudes toward conflict of interest in medical education and industry interactions.

	No/Don’t Know *n* (%)	Yes N (%)
**Knowledge of COI**		
Do you think you can define what a COI is?	112 (28.6)	280 (71.4)
Receiving a gift of minor value (book, pen, etc.) from the pharmaceutical industry	227 (57.9)	165 (42.1)
Having a close relative employed by the pharmaceutical industry	186 (47.4)	206 (52.6)
Being invited for lunch/dinner by the pharmaceutical industry	203 (51.8)	189 (48.2)
Participating in a training sponsored by the pharmaceutical industry	226 (57.7)	166 (42.3)
Being invited to a conference by the pharmaceutical industry	250 (63.8)	142 (36.2)
Participating in a clinical study paid by the pharmaceutical industry	169 (43.1)	223 (56.9)
Receiving a fellowship from the pharmaceutical industry	180 (45.9)	212 (54.1)
Being paid as a speaker by the pharmaceutical industry	164 (41.8)	228 (58.2)
Holding stock shares in the pharmaceutical industry	154 (39.3)	238 (60.7)
Receiving salary or honoraria from the pharmaceutical industry	142 (36.2)	250 (63.8)
**Mean knowledge score (SD)**	5.9 (3.1)	
**Attitudes Toward COI**		
Did you receive enough information about the declaration of COI during medical studies?	206 (52.6)	186 (47.4)
Did you receive a lecture or tutorial on COI during medical studies?	190 (48.5)	202 (51.5)
Did you do any personal research on the impact or declaration of COI?	246 (62.8)	146 (37.2)
Would you like to know the COI of your teachers when they teach you?	91 (23.2)	301 (76.8)
Do your teachers mention their COI during their lessons?	237 (60.5)	155 (39.5)
Do you feel adequately educated on COI issues in the medical field?	217 (55.4)	175 (44.6)
Should medical schools play a role in guiding student interactions with industry representatives?	62 (15.8)	330 (84.2)
Should the subject of COI be taught in medical school?	75 (19.1)	317 (80.9)
Would you like to receive more information regarding COI?	69 (17.6)	323 (82.4)
**Mean Attitude score (SD)**	5.4 (2.0)	

### Exposure to industry and perceived consequences

A significantly greater proportion of clinical students (*n = *211, 76.4%) than preclinical students (*n = *65, 23.6%) believed COI can induce bias in medical training (p = 0.009). Similarly, more clinical students (*n* = 210, 79.5%) than preclinical students (*n* = 54, 20.5%) agreed that COI can influence drug prescriptions (p < 0.001), and that COI can affect research (*n* = 216, 78.0% vs. *n* = 61, 22.0%, p < 0.001). No statistically significant differences were observed for other variables, including having met pharmaceutical representatives (p = 0.085), gift receipt (p = 0.984), attitudes toward COI in meals (p = 0.988), influence on future prescriptions (p = 0.194), informing patients (p = 0.775), and public COI declarations (p = 0.254). These findings highlight differences in COI perceptions and industry exposure across educational stages, as detailed in Table 3.

**Table 3:** Exposure to marketing strategies, potential consequences of COI for self and others, and transparency.

	Preclinical *n* (%) Yes	Clinical *n* (%) Yes	p-value
**Have you ever met a representative of the pharmaceutical industry?**	24 (34.8)	45 (65.2)	0.085
**Have you ever received a gift from the pharmaceutical industry?**	9 (26.5)	25 (73.5)	0.984
**COI can induce bias in medical training**	65 (23.6)	211 (76.4)	**0.009**
**COI can induce bias in drug prescriptions**	54 (20.5)	210 (79.5)	**<0.001**
**COI can induce bias in research**	61 (22.0)	216 (78.0)	**<0.001**
**Having received a gift will influence your future prescriptions**	16 (36.4)	28 (63.6)	0.194
**I consider it as a COI when attending a meal sponsored by the pharmaceutical industry**	12 (26.7)	33 (73.3)	0.988
**Patients should be informed of their physicians’ COI**	69 (28.0)	177 (72.0)	0.775
**I favor a public declaration of COI (e.g., Ministry of Health website)**	57 (25.3)	168 (74.7)	0.254

### Factors associated with COI knowledge and attitude

Regarding knowledge score, no significant differences were observed based on gender, university affiliation, or GPA (p = 0.411; p = 0.725 and p = 0.132, respectively). However, a significant difference was observed across educational stages (p = 0.001), where clinical students scored 6.17 ± 3.00 higher than preclinical students who scored 5.04 ± 3.05. While previous research experience and number of research publications did not reach statistical significance (p = 0.070 and p = 0.305, respectively), students previous research experience and higher number of publications demonstrated notably higher COI knowledge. The association between various factors and medical students’ knowledge about COI is summarized in Table 4.

**Table 4:** Association of different feature about knowledge of COI among medical students.

Variable	Response	Mean (SD)	p-value
**Gender**	Female	5.99 (3.12)	0.411^a^
	Male	5.74 (3.00)	
**University**	University of Jeddah	5.72 (3.14)	0.725^b^
	King Abdul Aziz University	6.08 (2.86)	
	King Saud University	6.25 (3.11)	
	Private Universities	5.79 (3.19)	
**Stage of Education**	Preclinical	5.40 (3.05)	***0.001*** ^a^
	Clinical	6.17 (2.00)	
**GPA**	Poor	3.50 (3.54)	0.132^b^
	Average	4.69 (2.75)	
	Good	6.05 (3.00)	
	Excellent	5.89 (3.12)	
**Research publication experience**	Previous research publication	6.21 (2.99)	0.070^a^
	No previous research	5.64 (3.09)	
**Research Publications**	1–3	6.06 (3.13)	0.305^b^
	4–6	7.12 (2.34)	
	7–10	5.50 (2.17)	
	More than 10	7.67 (1.86)	

(a) Independent T Test, (b) ANOVA, GPA: Grade Point Average.

Regarding attitude scores among participants, no significant impact was observed based on gender (p = 0.972), university affiliation (p = 0.198), or stage of education (p = 0.815). However, academic performance, as reflected by GPA, showed a strong association with better attitude scores (p < 0.001). Students with previous research experience demonstrated higher scores compared to those without research experience; however, this difference did not reach statistical significance (mean = 5.67, p = 0.073). Similarly, while the number of research publications was not significantly associated with higher attitude scores (p = 0.231), a positive trend was observed, with scores increasing progressively among students with more number publications. The association between various factors and medical students’ attitudes about COI is summarized in Table 5.

**Table 5:** Association of different feature towards attitude of COI among medical students.

Variable	Response	Mean (SD)	p-value
**Gender**	Female	5.44 (2.07)	0.972^a^
	Male	5.45 (2.01)	
**University**	University of Jeddah	5.28 (2.01)	0.198^b^
	King Abdul Aziz University	5.71 (2.05)	
	King Saud University	5.95 (1.73)	
	Private Universities	5.32 (2.14)	
**Stage of Education**	Preclinical	5.40 (2.08)	0.815^a^
	Clinical	5.46 (2.01)	
**GPA**	Poor	0.50 (0.71)	***<0.001*** ^b^
	Average	4.73 (1.95)	
	Good	5.38 (1.95)	
	Excellent	5.64 (2.05)	
**Research publication experience**	Previous research publication	5.67 (1.98)	0.073^a^
	No previous research	5.30 (2.05)	
**Research Publications**	1–3	5.55 (1.95)	0.231^b^
	4–6	5.94 (2.30)	
	7–10	6.00 (2.10)	
	More than 10	7.17 (1.47)	

(a) Independent T Test,

(b) ANOVA, GPA: Grade Point Average.

## Discussion

Conflict of interest has become an important topic in medical education, as it shapes the ethical and professional landscape in which future physicians are trained [14]. The findings of our study shed light on the current state of COI knowledge, attitudes, and practices among medical students.

One of the most prominent observations is that 71.4% of participants reported they could define COI, indicating a reasonable baseline awareness. This suggests that many medical students are at least somewhat familiar with the concept. However, our data also highlight that 28.6% still struggle with defining COI, reflecting a need for systematic and reinforced education. Similarly, Ji and Choe et al. found that 24.8% of respondents could define a COI, whereas Etain et al. reported a significantly higher rate of 64.6%. Interestingly, clinical students demonstrated higher knowledge scores than preclinical students. This trend is consistent with prior research, which shows that learners with greater clinical exposure or real-world experience are generally more aware of ethical and professional standards [15]. Morever, clinical students also showed a higher recognition of COI situations. Etain et al. similarly reported that clinical students had the highest levels of awareness, particularly regarding scenarios such as receiving salary or honoraria and holding stock shares (p < 0.0001) [11,12]. The clinical phase, in particular, often involves direct contact with industry representatives and the practical realities of prescribing, which may explain the highest knowledge scores in that group.

Despite this increasing awareness among advanced learners, there remains a sizable fraction of participants who do not view certain industry interactions as potential conflicts. Nearly 58% did not consider receiving minor gifts a conflict, and many were unconcerned with lunch/dinner invitations. Yet, research suggests even small gifts may often subtly affect prescribing behavior and judgment in clinical practice. Field and Lo et al. shows that although small gifts to physicians may seem to be inconsequential, some research suggests that small gifts can contribute to unconscious bias in decision making and advice given [14]. Another study by Andresen et al. shows that both medical students and faculty indicated that is unacceptable for medical students or faculty to accept gifts from pharmaceutical or medical device representatives [16]. The psychological principle of reciprocity implies that receiving something of value, even if small, can create an unconscious sense of obligation [17]. The fact that many participants do not see these gifts as problematic suggests that more nuanced education is needed, clarifying how even minor gestures can shape clinical decision-making.

On a more positive note, most participants recognized that involvement in industry-sponsored clinical studies, receiving speaker fees, or holding stock in pharmaceutical companies can pose clear conflicts. Such recognition reflects growing awareness that direct financial ties are among the most overt forms of COI. These results are consistent with studies in other countries like Korea that have reported students and physicians alike perceiving major financial stakes—like research funding or speaker honoraria—as high-risk areas for bias [18]. Encouragingly, this indicates that while small gifts remain controversial, the line is clearer for more substantial forms of industry engagement.

In terms of attitudes, our findings reveal that around half of the students did not feel they had received sufficient education on COI, and close to half had never attended a lecture or tutorial on the topic. These statistics resonate with other global data: many medical curricula either do not address COI in depth or do so sporadically [19]. A majority in our study believed that medical schools should play a guiding role in shaping student-industry interactions, and over 80% wanted COI included in the curriculum. This is a crucial point, as prior research indicates that formal COI policies and dedicated educational sessions can improve both the knowledge and ethical decision-making of future physicians [20]. The high demand for more comprehensive COI education suggests a gap between students’ perceived needs and what they are currently receiving in their training.

The association of higher GPA with a more developed and positive attitude toward COI is an intriguing outcome. Students with a poor GPA had markedly lower attitude scores, possibly reflecting less engagement with academic content overall, including ethical issues. Similarly, Previous studies indicate that GPA can be a predictor of academic integrity. Students with higher GPAs tend to demonstrate greater adherence to ethical practices in their academic work. Conversely, those experiencing stress or performance pressure may be more prone to engaging in dishonest behavior [21].

Meanwhile, those with more research experience, especially those who had published multiple papers, tended to show higher knowledge and more robust attitudes. This aligns with previous literature indicating that involvement in research can sharpen awareness of ethical considerations, as learners often encounter discussions of funding, authorship, and bias during the research process [22]. Even though our study found this association to be a trend rather than a statistically significant difference, it still points to the value of hands-on scholarly activities in reinforcing ethical principles. Thus, preclinical students often indicated low or uncertain monetary thresholds for COI, whereas clinical students reported significantly higher cutoffs, suggesting differences in exposure and potentially inconsistent teaching or unclear institutional policies.

Although our study did not differentiate between direct and indirect COI, this conceptual distinction is crucial in the context of medical ethics and education. Direct COIs typically involve explicit financial relationships, such as receiving gifts, fees, or funding from industry stakeholders. Indirect COIs, on the other hand, may arise from more subtle influences, such as career advancement, reputational benefits, or institutional pressures [14,23]. These indirect forms of COI can be insidious, as they are often less visible but may still exert significant influence on clinical judgment, academic decisions, and teaching content. Future research should explore whether medical students are able to recognize and navigate both types of COI, and whether current curricula effectively distinguish between them.

This study has some limitations, including its and reliance on self-reported data, which may be subject to recall and social desirability bias. Additionally, the sample is restricted to a specific region, limiting generalizability of the findings to other areas or medical institutions with different curricula or policies. The absence of longitudinal follow-up restricts insights into evolving COI perceptions over time, warranting further research. Future studies should explore longitudinal assessments of COI education, intervention-based approaches to improve knowledge, cross-cultural comparisons, and the impact of faculty and industry interactions on students’ perceptions and ethical decision-making.

## Conclusion

Our findings indicate a growing awareness of COI among students, yet notable gaps in knowledge and attitudes remain. Many students are eager to learn more and seek institutional support for formal COI education. This aligns with broader medical education literature, emphasizing the need to integrate COI topics early and continuously into curricula. Structured education through lectures, tutorials, and transparent discussions about faculty COI can foster robust ethical frameworks, enhance academic integrity, and nurture a professional identity that prioritizes patient welfare, ultimately leading to more trustworthy healthcare practices.

## Supporting information

S1 Text Survey questionnaire used to assess knowledge and attitudes of medical students regarding conflict of interest. (PDF)
