# Supplementary material for: The hidden influence: Medical students’ knowledge and attitude of conflict of interest–A cross-sectional study in Jeddah, Saudi Arabia
Source: PLoS One. 2025 Aug 1;20(8):e0328884. doi: 10.1371/journal.pone.0328884 (PMC12316206; doi:10.1371/journal.pone.0328884)
Supplement: S1 Text — (PDF) [file pone.0328884.s001.pdf]

# Questionnaire: Conflict of interest

**I consent to participate in this survey and understand that my responses will be used for research purposes.**

Agree
Disagree (out of questionnaire if click)

SECTION 1: General Personal Information

1.1-Age
Written text

1.2-Gender
Male/ female

1.3- university *

King Abdulaziz University
University of Jeddah
King Saud bin Abdulaziz University for Health Sciences - Jeddah
Batterjee Medical College
Ibn Sina National College for Medical Studies
Fakeeh College of Medical Sciences
Other:

1.4-Academic year*
Pre-clinical years
Clinical years

1.5- GPA out of 5 (GPA out of 4)
4.5 - 5 (3.5 - 4)
3.75 - 4.49 (2.75 - 3.49)
2.75 - 3.74 (1.75 - 2.74)
2 - 2.74 (1 - 1.74)

have you ever published any research before?
Yes > go to next question
No

>>> if yes, how many?

No previous research
1 to 3
4 to 6
7 to 10
More than 10

**SECTION 2: Knowledge of Conflicts of Interest and Public Interest Disclosure**

**2.1. Do you think you can define what a conflict of interest is?**
**Yes / No / I'm not sure**

2.2 Do you consider the following situation as a conflict of interest?
(yes – no – I don't know)

Set 1

Receiving a gift of minor value (book, pen, etc.) from the pharmaceutical industry
Having a close relative employed by the pharmaceutical industry
Being invited for lunch/dinner by the pharmaceutical industry
Participating in a training sponsored by the pharmaceutical industry
Being invited to a conference by the pharmaceutical industry
Set 2

Participating in a clinical study paid by the pharmaceutical industry
Receiving a fellowship from the pharmaceutical industry
Being paid as a speaker by the pharmaceutical industry
Holding stock shares in the pharmaceutical industry
Receiving salary or honoraria from the pharmaceutical industry

**SECTION 4: Your Opinion on Conflicts of Interest and Based on Your Personal Experience**

**Can you answer the following questions?**

- **Exposure to marketing strategies**

-Have you ever met a representative of the pharmaceutical industry?
> I do not wish to respond / No / Yes

-Have you ever received a gift from the pharmaceutical industry?
I do not wish to respond / No / Yes

- **Consequences of COI for others**

-COI can induce bias in medical training
(yes/ no/ I don’t know)
-COI can induce bias in drug prescriptions
(yes/ no/ I don’t know)
-COI can induce bias in research
(yes/no/ I don’t know)

- **Self-consequences of COI**

-Having received a gift will influence your future prescriptions
>(I do not accept or receive gifts from a pharmaceutical company / I'm uncertain / No / Yes)

-I consider it as a COI when attending a meal sponsored by the pharmaceutical industry
>I refuse to attend such meetings / I don't know / No / Yes

- **Transparency**

-Patients should be informed of their physicians’ COI
(yes/no/ I don’t know)
-I favor a public declaration of COI (e.g., Ministry of Health website)
(yes/no/ I don’t know)

**SECTION 3: Information Received During Your Studies on Conflicts of Interest (COI)**

**For the remainder of the survey, a conflict of interest will be defined as follows: "A conflict of interest occurs when the decisions made by a physician, researcher, or expert in a crucial area, such as patient well-being, research integrity, or the validity of a recommendation, are at significant risk of being**

**compromised by a competing objective, such as financial gain, reputation, or the ability to raise research funds**

3.2. During your medical studies, do you feel that you received enough information about the declaration of COI?
yes / no

3.3. During your medical studies, did you receive a lecture or a tutorial on COI?

yes / no

3.4. During your medical studies, did you do any personal research (internet for example) on the impact of COI or on the declaration of COI?
yes / no

4.6. I would like to know the COI of my teachers when they teach me? (
yes / no

4.5. Do your teachers mention their COI during their lessons?
yes / no

**Do you feel adequately educated on conflict of interest (COI) issues in the medical field?**
Yes/no

**Do you believe that medical schools should play a role in guiding student interactions with industry representatives?**
Yes / No

3.8. Do you think that the subject of Conflicts of Interest should be taught in medical school?
- Yes / No

3.9. Would you like to receive more information regarding Conflicts of Interest?
- Yes / No

3.7. In your opinion, how should information on conflicts of interest be provided?
- Lecture / Tutorial
- Course integrated into Critical Appraisal of Articles (CAA)
- Information provided outside of the faculty (conferences/meetings)
- Newsletter or mailing list
- Written document sent to each student
- Other; free text field
- No need to be provided
